# Plasma level of advanced oxidation protein products as a novel biomarker of acute lung injury following cardiac surgery

**DOI:** 10.1186/s40064-016-1899-9

**Published:** 2016-02-29

**Authors:** Songlin Du, Jun Ai, Xiangzhen Zeng, Jun Wan, Xu Wu, Jianxing He

**Affiliations:** Department of Thoracic and Cardiovascular Surgery, Nanfang Hospital of Southern Medical University, 1838 North Guangzhou Avenue, Guangzhou, 510515 China; Division of Nephrology, Nanfang Hospital of Southern Medical University, Guangzhou, 510515 China; Division of Nephrology, Loudi City Central Hospital, Loudi, 417099 China; State Key Laboratory of Respiratory Disease, Guangzhou, 510120 China; National Clinical Research Center for Respiratory Disease, Guangzhou, 510120 China

**Keywords:** Acute lung injury, Cardiopulmonary bypass, Advanced oxidation protein products, Oxidative stress, Biomarker

## Abstract

This study was designed to test the hypothesis that whether the plasma level of advanced oxidant protein products (AOPPs) would be useful for the clinical diagnosis of acute lung injury (ALI) following cardiac surgery with the technique of cardiopulmonary bypass (CPB). In this prospective study, seventy consecutive adults undergoing open heart surgery with CPB were included and assigned into the ALI (n = 18) and non-ALI (n = 52) groups according to the American-European Consensus Criteria. Plasma concentrations of AOPPs were measured at baseline, postoperative 1 h, 12 h, 24 h, and 48 h. Eighteen patients (25.7 %) developed ALI after surgery. The plasma levels of AOPPs in the ALI group were significantly increased and remained considerably higher at all time points after operation (all *P* < 0.05). Multivariate logistic regression analysis revealed that the plasma level of AOPPs at 1 h after operation was an independent predictor for the diagnosis of ALI (OR 1.164; 95 % CI 1.068–1.269; *P* = 0.001). Plasma level of AOPPs could serve as an early biomarker of the incidence of ALI in adult patients who underwent open cardiac surgery with the technique of CPB.

## Background

Acute lung injury (ALI) is a common complication in patients undergoing cardiac surgery with the technique of cardiopulmonary bypass (CPB) with a high morbidity and mortality (Vlaar et al. 2011; Apostolakis et al. 2010; Koch et al. 2009; Stephens et al. 2013). If ALI evolved into acute respiratory distress syndrome (ARDS), the mortality of ALI may be as high as 80 % (Koch et al. 2009; Stephens et al. 2013; Ng et al. 2002). ALI complication could prolong the length of intensive care unit (ICU) and hospital stay, and raise treatment expenses (Kogan et al. 2014). In spite of widespread studies, no effective therapy can accelerate the recovery. Predicting ALI/ARDS would be extremely valuable for making clinical decisions and guiding subsequent research.

The incidence of ALI/ARDS following CPB was associated with multiple overlapping biological pathways, cell and tissue injury, which may generate a large quantity of potential biomarkers (Levitt et al. 2009). Ideal biomarkers could be used to identify pivotal biological pathways underlying the pathogenesis of ALI/ARDS and played a vital role in the early diagnosis of this disorder. Unfortunately, biomarkers remained largely a research tool over the past two decades. Previous studies have demonstrated that oxidative stress contributes to the progression of ALI/ARDS induced by different causes, such as CPB, sepsis, trauma and multiple transfusions, etc. (Uchida et al.^2006^; Calfee et al. 2008; Chow et al. 2003). Pulmonary oxidant production can lead to the incidence of ALI and occasionally progressive lung injury (Chow et al. 2003; Ward 2010). One of the pivotal mechanisms of ALI/ARDS after CPB is oxidative injury to the lung mediated by reactive oxygen species (ROS) including superoxide anion radical (O_2_^−^), hydrogen peroxide (H_2_O_2_), hydroxyl radical (OH^−^), and hypochlorous acid (HOCl) (Ward 2010). ROS can lead to cell injury via protein oxidation that alters protein activity, causing release of proteases and inactivation of antioxidant and anti-protease enzymes (Chow et al. 2003; Ward 2010; Tuinman et al. 2013). Accumulated experimental evidence supported that HOCl may lead to irreversible lung damage and even fibrosis (Chow et al. 2003; Ward 2010; Tuinman et al. 2013; Johnson and Koval 2009). Advanced oxidation protein products (AOPPs) are generated as a result of the reaction between plasma proteins and chlorinated oxidants, such as chloramines and HOCl (Wu et al. 2015). More importantly, AOPPs are utilized not only as a biomarker of oxidative stress but also a mediator of inflammation, which potentially reflects multiple aspects of the pathogenesis and pathophysiology of ALI/ARDS following CPB (Liang et al. 2012; Gangemi et al. 2015; Bochi et al. 2014). These modified molecules may efficiently monitor the progression and outcome of ALI/ARDS induced by CPB. Hence, we hypothesized that AOPPs would be useful for biological confirmation of the clinical diagnosis of ALI/ARDS following cardiac surgery with CPB.

The aim of this prospective study was to examine the plasma levels of AOPPs in patients with ALI following cardiac surgery and explore whether these molecules serve as useful markers in predicting the incidence of ALI in these patients.

## Methods

### Study population

This prospective study was performed in Nanfang Hospital affiliated to Southern Medical University, China. Inclusion criteria should be met if seventy patients aged >18 years undergoing cardiac surgery with CPB at our department between January and May 2014 were included in this clinical trial. The study protocols were approved by the Ethics Committee of Nanfang Hospital of Southern Medical University. Informed consents were obtained from all participants included in this study. Exclusion criteria: Patients were excluded if they were with abnormal liver or renal function, pulmonary inflammation before surgery, pulmonary edema due to cardiac dysfunction or died from cardiac dysfunction.

### Data collection

Demographic data, preoperative risk factors, and perioperative surgical and anesthetic management data including the patient’s gender, age, body mass index (BMI), pulse oximetry saturation (SpO_2_), CPB time and Clamp time and arterial partial pressure of oxygen to fraction of inspired oxygen(PaO_2_/FiO_2_), length of surgery intensive care unit (SICU) stay and hospital stay were collected. A pulmonary artery catheter was inserted into all patients before operation. CPB was performed under mild to moderate hypothermia (28–34 °C) in all patients. Chest radiograph was routinely taken before surgery and every day after surgery. Echocardiography was performed routinely to evaluate postoperative cardiac function at any time if necessary. Left ventricular function was evaluated by the index of ejection fraction (EF).

### Main definitions

Patients were categorized into the ALI (n = 18) and non-ALI groups (n = 52) according to American-European Consensus Conference (AECC) definition (Bernard et al. 1994). ALI was defined as follows: oxygenation: PaO_2_/FiO_2_ <300 mmHg (regardless of positive end-expiratory pressure); chest radiograph: bilateral infiltrates seen on frontal chest radiograph, pulmonary artery occlusion pressure:<18 mmHg, the absence of cardiogenic pulmonary edema (CPE). CPE was identified when the pulmonary arterial occlusion pressure was >18 mmHg or by the presence of central venous pressure >14 mmHg and left ventricular EF < 45 %.

### Determination of plasma AOPPs

For each patient, serial plasma samples were obtained at the baseline level, 1, 12, 24, and 48 h after operation and stored at −80 °C. In previous reports, plasma AOPPs were determined using the semi-automated method (Lentini et al. 2010). In test wells, a portion of 200 μL of plasma was diluted to a ratio of 1:5 in phosphate-buffered saline (PBS), placed into a 96-well microtiter plate, and supplemented with 20 μL of acetic acid. In standard wells, 10 μL of potassium iodide was added to 200 μL of chloramine-T solution (0–100 μmol/L) and followed by 20 μL of acetic acid. The absorbance value was immediately measured at a wavelength of 340 nm. The concentration of AOPPs was expressed in unit of μmol/L of chloramine-T equivalents. Laboratory operators were blinded to the ALI patients, and relevant investigators involved in the interpretation of ALI were blinded to AOPPs levels.

### Statistical analysis

All statistical analyses were performed with SPSS 19.0 for Windows (SPSS Inc., Chicago, IL, USA). Continuous data were analyzed for normal distribution with one-sample Kolmogorov–Smirnov test. Normally-distributed data were further analyzed using unpaired student’s *t* test. Abnormally-distributed data were analyzed using Mann–Whitney U test. χ^2^ test was employed to compare categorical data as appropriate. A multivariate analysis of variance procedure (MANOVA) was used to assess whether a significant difference existed in the assessment of AOPPs levels between two groups. Receiver operating characteristic curve (ROC) was computed, and area under the curve (AUC) was calculated to evaluate the significance of these biomarkers in diagnosing ALI. Risk factors related to the development of ALI, such as age, sex, body mass index (BMI), CPB time or clamp time, were introduced into the univariate analysis. Multivariate stepwise forward logistic regression analysis was then performed for statistically significant univariate predictors to determine the independent risk factor for ALI after CPB. *P* < 0.05 was accepted as statistically significant.

## Results

### Demographic characteristics

**Fig. 1:**
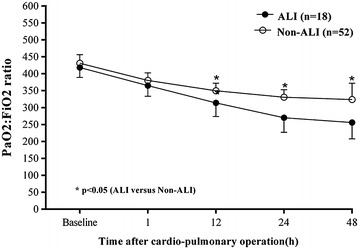
The ratio of PaO_2_ and FiO_2_ at baseline level and different time points after operation. *Error bar* are SD

A total of 70 patients fulfilled the inclusion criteria. Among them, 18 (25.7 %) developed ALI. Demographic and perioperative characteristics of all participants are illustrated in Table 1. Age, BMI, smoking, diabetes mellitus, cardiothoracic ratio, LVEF, operation type, CPB time, clamp time and preoperative AOPPs did not significantly differ between two groups. However, the incidence of COPD, hypertension and the proportion of male were significantly increased in patients with ALI compared with non-ALI counterparts. In addition, the duration of SICU stay and hospital stay were significantly prolonged in the ALI group compared with those in the non-ALI group. The first significant decline of PaO_2_:FiO_2_ ratio occurred at 1 h after operation (Fig. 1). The ratio of PaO_2_:FiO_2_ at postoperative 12, 24 and 48 h in the ALI group was significantly lower than that in the non-ALI group, as illustrated in Table 2 and Fig. 1. Interestingly, PaO_2_:FiO_2_ ratio in the ALI group decreased to lowered than 300 (270 ± 43.1) at 24 h after operation.

**Table 1:** Demographic and clinical characteristics between ALI and non-ALI patients

	ALL (n = 70)	ALI (n = 18)	Non-ALI (n = 52)	P value
Basic parameters				
Male (n)	37	12	25	<0.001
Age (years)	47.9 ± 11.3	57.7 ± 11.6	46.6 ± 11.4	0.105
Body mass index	21.6 ± 3.60	22.9 ± 2.90	21.1 ± 3.8	0.081
Smoking (n)	15	6	9	0.153
COPD (n)	7	5	2	0.01
Hypertension (n)	9	6	3	0.007
Diabetes mellitus (n)	4	3	1	0.05
Cardiothoracic ratio	0.61 ± 0.09	0.63 ± 0.08	0.61 ± 0.1	0.415
LVEF(%)	59.9 ± 7.0	57.9 ± 6.5	60.6 ± 7.1	0.154
Intraoperative parameters				
Type of surgery				0.223
Coronary artery disease (%)	11 (15.7 %)	5 (27.8 %)	6 (11.5 %)	
Valve heart disease (%)	52 (74.3 %)	12 (66.7 %)	40 (77.0 %)	
Congenital heart disease (%)	7 (10 %)	1 (5.5 %)	6 (11.5 %)	
CPB time (min)	106.4 ± 37.8	116.3 ± 31.5	102.9 ± 39.4	0.198
Clamp time (min)	68.5 ± 30.8	73.3 ± 24	66.9 ± 32.9	0.452
Peri-operative risk factors				
Preoperative AOPPs (μmol/L)	46.0 ± 10.5	47.1 ± 7.9	44.8 ± 10.8	0.394
SICU stay (days)	3 (2, 5)	7.3 ± 4.4	3 (2, 3)	<0.001
Hospital stay (days)	17.3 ± 5.9	24.4 ± 5.8	14.8 ± 3.4	<0.001

Data are presented as number of patients (%), mean ± SD, or counts, as appropriate

*ALI* acute lung injury, *COPD* chronic obstructive pulmonary disease, *LVEF* left ventricular ejection fraction, *CPB* cardiopulmonary bypass, *SICU* surgery intensive care unit

**Table 2:** The ratio of PaO_2_ and FiO_2_ at baseline level and different time points after operation

PaO_2_:FiO_2_ ratio	Before operation	1 h after operation	12 h after operation	24 h after operation	48 h after operation
ALI	418 ± 29.5	365 ± 31.6	314 ± 40.2^a^	270 ± 43.1^a^	256 ± 48.2^a^
Non-ALI	431 ± 25.4	380 ± 21.9	350 ± 22.0	331 ± 22.0	324 ± 47.7

Data were shown as mean ± SD

*ALI* acute lung injury

^a^P < 0.05 versus Non-ALI group at the same time point

### Plasma AOPPs

**Fig. 2:**
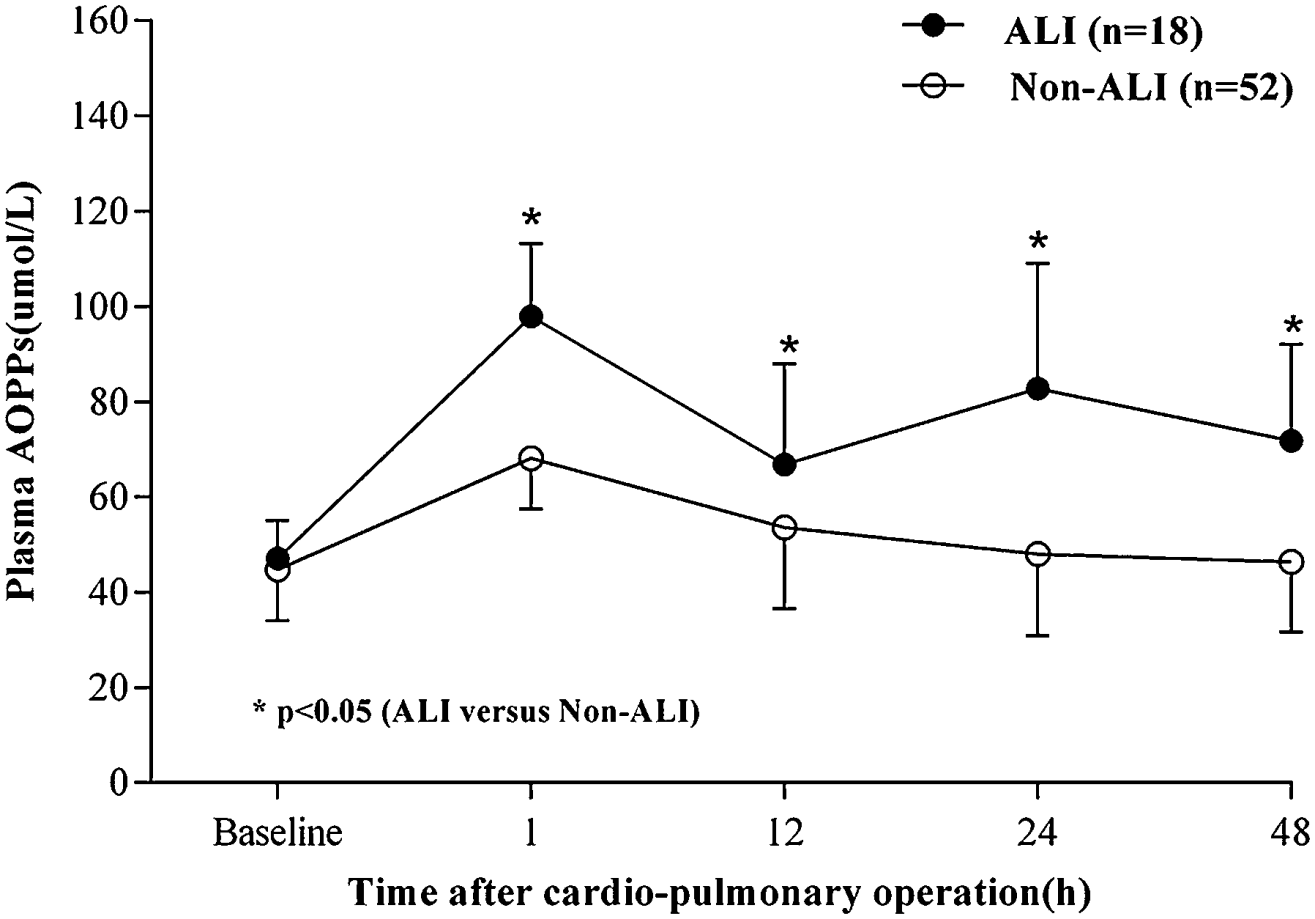
Mean plasma concentration of AOPPs at baseline level and different time points after operation. *Error bar* are SD

For all patients, postoperative plasma levels of AOPPs were increased compared to the baseline level (Fig. 2). Plasma concentration of AOPPs was shown in Table 3. The levels of plasma AOPPs were first significantly increased at 1 h after operation and declined at postoperative 12 h in all patients. The levels of plasma AOPPs in the ALI group were significantly higher than those in the non-ALI group at 1, 12, 24 and 48 h after operation. Plasma level of AOPPs in non-ALI patients decreased to the baseline level, whereas those in ALI counterparts remained higher than the baseline level at 24 h after operation (Table 3; Fig. 2). In the ALI group, the level of AOPPs peaked at postoperative 1 h, dramatically decreased at 12 h after operation, and began to increase at postoperative 24 h after operation.

**Table 3:** Plasma AOPPs (μmol/L) at baseline level and different time points after operation

AOPPs	Before operation	1 h after operation	12 h after operation	24 h after operation	48 h after operation
ALI	55.7 ± 11.3	97.9 ± 15.3^a^	66.8 ± 21.2^a^	82.8 ± 26.2^a^	71.8 ± 20.2^a^
Non-ALI	44.8 ± 10.8	68.2 ± 10.7	53.6 ± 17.1	48.0 ± 17.1	46.4 ± 14.7

Data were shown as mean ± SD

*ALI* acute lung injury

^a^P < 0.05 versus Non-ALI group at the same time point

### Plasma AOPPs as a predictor for ALI progression

Univariate analysis demonstrated that plasma level of AOPPs at 1 h after operation (OR 1.148; 95 % CI 1.065–1.237; *P* < 0.001), age (OR 1.103; 95 % CI 1.041–1.168; *P* = 0.001), COPD (OR 9.615; 95 % CI 1.671–55.314; *P* = 0.011), hypertension (OR 8.167; 95 % CI 1.781–37.449; *P* = 0.007) were significantly associated with increased risk of ALI.

**Fig. 3:**
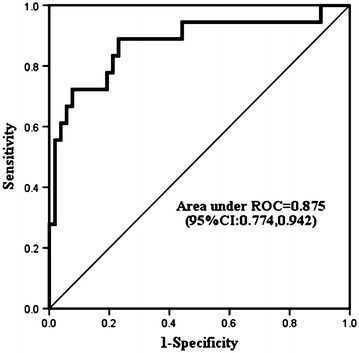
ROC curve analysis of plasma levels of AOPPs measured at 1 h after operation. The area under the curve was 0.875 (95 % CI 0.774, 0.942)

After adjustment for age, diabetes mellitus and hypertension by multivariable logistic analysis, plasma level of AOPPs at 1 h after operation (OR 1.1674; 95 % CI 1.068–1.269; *P* = 0.001) and COPD (OR 28.706; 95 % CI 1.770–465.640; *P* = 0.018) were highly associated with increased risk of ALI (Table 4). As Fig. 3 illustrated, the area under the ROC curve of plasma level of AOPPs at postoperative 1 h was calculated as 0.875, indicating the clinical significance of plasma level of AOPPs at 1 h after operation in the diagnosis of ALI. Derived sensitivity, specificity, and predictive cutoff value of plasma level of AOPPs at postoperative 1 h are listed in Table 5. Plasma level of AOPPs at 1 h after operation yielded the highest sensitivity and specificity at the cutoff value of 81.89 μmol/L (Table 5).

**Table 4:** Univariate and multivariate analysis of plasma AOPPs after operation for prediction of ALI

	Univariate		Multivariate	
	OR (95 % CI)	P	OR (95 % CI)	P
AOPPs 1 h after operation	1.148 (1.065, 1.237)	<0.001	1.164 (1.068, 1.269)	0.001
Age	1.103 (1.041, 1.168)	0.001	1.054 (0.984, 1.129)	0.130
COPD	9.615 (1.671, 55.314)	0.011	28.706 (1.770, 465.64)	0.018
Diabetes mellitus	10.20 (0.987, 105.38)	0.051	1.485 (0.085, 26.040)	0.787
Hypertension	8.167 (1.781, 37.449)	0.007	0.665 (0.059, 7.501)	0.741
BMI	1.147 (0.984, 1.336)	0.079	0.998 (0.758, 1.313)	0.989
Systolic BP	1.049 (1.001, 1.099)	0.052	1.061 (0.982, 1.147)	0.133

P < 0.10 in the univariate analysis were candidates for the multivariable logistic analysis (including variables: AOPPs 1 h after operation, Age, COPD, diabetes mellitus, hypertension, BMI and systolic BP)

*ALI* acute lung injury, *Systolic BP* systolic blood pressure

**Table 5:** Performance of plasma AOPPs 1 h after operation (μmol/L) for diagnosis of ALI

	Area under ROC curve (95 % CI)	At cutoff value of 81.89 (μmol/L)			
		Sensitivity (95 % CI)	Specificity (95 % CI)	PPV (95 % CI)	NPV (95 % CI)
AOPPs 1 h after operation (μmol/L)	0.875 (0.774, 0.942)	0.722 (0.465, 0.903)	0.923 (0.815, 0.979)	0.765 (0.492, 0.935)	0.906 (0.793, 0.969)

## Discussion

It is well known that the production of oxidants within the lung can lead to the incidence of ALI, reversible or even irreversible progressive lung injury (Ward 2010; Johnson and Koval 2009). Reversibility occurs when the stimulus of intrapulmonary oxidants overcomes the limit of natural antioxidant enzymes in the lung. Stress-induced alterations in the conformation or structure of proteins are capable of inducing protein dysfunction or inhibiting protein degradation (Chow et al. 2003). Modified proteins can subsequently induce cellular dysfunctions and tissue damage. Under such conditions, the regenerative capacity of the lung is overwhelmed, which promotes the development of ALI/ARDS (Apostolakis et al. 2010). AOPPs are relatively stable molecules that can be measured by fast and inexpensive techniques, which makes AOPPs as suitable biomarkers for monitoring oxidative stress (Bochi et al. 2014). Previous studies provided evidence that the levels of oxidative stress in patients receiving off-pump operations were significantly lower compared with those in the counterparts undergoing CPB surgery (Matata et al. 2000). In this investigation, plasma AOPPs levels were elevated after cardiac surgery with CPB and directly associated with the occurrence of ALI and a poor clinical outcome, validating the hypothesis that plasma AOPPs is a reliable biomarker of the risk of ALI following cardiac surgery. Furthermore, the AOPPs levels in the ALI group were significantly higher than those in the non-ALI group at 1, 12, 24 and 48 h after operation, suggesting that plasma AOPPs levels may serve as a reliable predictor for the progression of ALI following CPB in adult patients.

Dynamic detection of the plasma levels of AOPPs was performed at 1, 12, 24 and 48 h. The plasma levels of AOPPs in all patients peaked quickly at 1 h after operation, and then dropped to the baseline level at postoperative 12 h. At 24 h after surgery, the levels of plasma AOPPs in the ALI patients increased again and were maintained significantly higher compared with those in the non-ALI counterparts. The elevation in the plasma AOPPs levels may reflect the duration of excessive oxidative stress. Meanwhile, PaO_2_:FiO_2_ ratio decreased at 12 h after operation, and then declined to <300, which was considered as the diagnostic level of ALI at postoperative 24 h. Thus, plasma levels of AOPPs at 1 h after operation served as the first predictor of the incidence of ALI. The cutoff value of AOPPs was calculated as 81.89 μmol/L. There is growing evidence that increased oxidative stress ultimately induces endogenous defense mechanisms and inhibitory responses through negatively modulating cellular signaling pathways (Ward 2010). Understanding the dynamic interaction between intrapulmonary oxidant production and engagement of antioxidant defenses provides supplementary information relevant to the incidence of ALI/ARDS following CPB.

Several potential limitations have to be acknowledged in our study. First, the results were obtained based on a relatively small sample size in one single center. Hence, a larger-cohort study is needed to verify these results. Second, traditional inflammatory cytokines were not quantitatively measured in this study. However, none of them was a specific biomarker for the occurrence of ALI post-CPB. Third, it is difficult to conclude that whether increased plasma levels of AOPPs are the cause of ALI or the consequence of major surgery which subsequently leads to ALI. However, due to the fact that perioperative characteristics did not significantly differ between the ALI and non-ALI groups, we believe that elevated plasma levels of AOPPs serve as a marker for the incidence after CPB. Specifically-designed study is required to further address this issue.

## Conclusions

In conclusion, this is the first study identifying the potential role of AOPPs involved with the incidence of ALI following open cardiac surgery with the technique of CPB. We found that plasma levels of AOPPs could be used as an early biomarker of the occurrence of ALI after cardiac surgery with CPB in adult patients. The plasma levels of AOPPs are associated with lung function and clinical outcome after CPB. The precise role of AOPPs in the clinical presentations and progression of ALI following cardiac surgery remain to be elucidated.

## Abbreviations

AOPPs: advanced oxidant protein products
ALI: acute lung injury
CPB: cardiopulmonary bypass
ARDS: acute respiratory distress syndrome
ICU: intensive care unit
ROS: reactive oxygen species
HOCl: hypochlorous acid
BMI: body mass index
SpO_2_: pulse oximetry saturation
PaO_2_/FiO_2_: arterial partial pressure of oxygen to fraction of inspired oxygen
SICU: surgery intensive care unit
LVEF: left ventricular ejection fraction
COPD: chronic obstructive pulmonary disease
PBS: phosphate-buffered saline
ROC: receiver operating characteristic curve
AUC: area under the curve
